# A modified IRS-III chemotherapy regimen leads to prolonged survival in children with embryonal tumor with multilayer rosettes

**DOI:** 10.1093/noajnl/vdaa120

**Published:** 2020-09-18

**Authors:** Derek Hanson, Lindsey M Hoffman, Sumanth Nagabushan, Liliana C Goumnerova, Allison Rathmann, Timothy Vogel, David S Ziegler, Susan Chi

**Affiliations:** 1 Department of Pediatrics, Hackensack Meridian School of Medicine at Seton Hall University, Nutley, New Jersey, USA; 2 Department of Pediatrics, Joseph M. Sanzari Children’s Hospital, Hackensack University Medical Center, Hackensack, New Jersey, USA; 3 Center for Discovery and Innovation, Hackensack Meridian Health, Nutley, New Jersey, USA; 4 Center for Cancer and Blood Disorders, Phoenix Children’s Hospital, Phoenix, Arizona, USA; 5 Sydney Children’s Hospital, Randwick, New South Wales, Australia; 6 University of New South Wales, Sydney, New South Wales, Australia; 7 Department of Neurosurgery, Hackensack University Medical Center, Hackensack, New Jersey, USA; 8 Morristown Medical Center, Morristown, New Jersey, USA; 9 Saint Peters University Hospital, New Brunswick, New Jersey, USA; 10 Dana-Farber Cancer Institute, Boston, Massachusetts, USA; 11 Harvard Medical School, Boston, Massachusetts, USA; 12 Trombo Protea, Inc., Weston, Massachusetts, USA

**Keywords:** brain tumor, chemotherapy, embryonal tumor with multilayer rosettes, pediatric

## Abstract

**Background:**

Embryonal tumor with multilayer rosettes (ETMR) is a rare CNS malignancy affecting young children that carries a very poor prognosis. Treatment with intensive surgical resection, radiotherapy, and high-dose chemotherapy is insufficient treatment in the vast majority of cases. Effective, biologically based therapies for this tumor are therefore desperately needed. The Dana-Farber Cancer Institute–modified IRS-III protocol incorporates preclinically active agents, such as doxorubicin and actinomycin D, into the treatment regimen for ETMR and may improve patient outcomes.

**Methods:**

The authors present a case series of 5 children with ETMR treated with an IRS-III-based chemotherapy backbone.

**Results:**

All 5 patients received a gross-total tumor resection. Patients received between 12 and 51 weeks of IRS-III therapy at the discretion of their treating physician. Four patients received focal radiation therapy, with the fifth patient instead receiving a cycle of high-dose chemotherapy with autologous stem cell rescue. Four patients have progression-free survival of more than 18 months. Chemotherapy treatment was reasonably tolerated by all 5 patients with one case of mild sinusoidal obstructive syndrome and one case of Grade 3 peripheral neuropathy.

**Conclusions:**

The patient outcomes in this small cohort are far better than would be expected based on the historical survival for this tumor. Given the tremendous need for effective therapy for ETMR, further investigation of this approach is warranted. An international consensus protocol based on the IRS-III regimen has been developed and will be available through a multicenter clinical trial and a global treatment registry.

**Key Points**

- Chemotherapy with preclinically active agents may improve outcomes for ETMR.
- IRS-III-based treatment led to progression-free survival of more than 18 months in 4 of 5 patients with ETMR.

**Importance of the Study**

ETMR is an aggressive pediatric CNS tumor for which there is currently no standard of care. Traditional approaches for infant brain tumors have been largely ineffective, and improved treatments incorporating the known biology of ETMR must be pursued. The Dana-Farber Cancer Institute–modified IRS-III protocol provides an avenue for incorporating preclinically active agents into therapy. A series of 5 children treated with this regimen demonstrates promising results with 4 patients achieving progression-free survival of more than 18 months. Based on the available preclinical data and these encouraging clinical outcomes, a global clinical investigation for ETMR has been developed.

Embryonal tumor with multilayer rosettes (ETMR) is a rare and highly aggressive CNS neoplasm that occurs almost exclusively in young children and is associated with an extremely poor prognosis. There is no current standard therapy for the tumor and survival is historically dismal even with intensive therapy. The Dana-Farber Cancer Institute–modified IRS-III protocol (DFCI-IRS-III) provides an avenue for incorporating preclinically active agents into therapy and may improve outcomes for ETMR. Preliminary efficacy of the regimen has been demonstrated in a small cohort of patients with long-term survival and will be explored further in a larger clinical investigation.

ETMR almost always arises in children under 4 years of age, with a median age of diagnosis of approximately 2.5 years.^1,2^ Two thirds of tumors are located supratentorially and have predilection for the fronto-parietal region. The remainder occur in the cerebellum, brainstem, and rarely, the spinal cord.^1–3^ The majority of patients with ETMR present with localized disease, though approximately 20% are metastatic at diagnosis.^1,2^ Clinically, ETMR follows an extremely aggressive course with many patients experiencing tumor progression during therapy or shortly after completing treatment. Korshunov et al. published a series of 55 children with ETMR, which underscored the dismal prognosis of this tumor. Fifty of the described patients experienced disease recurrence with a median progression-free survival (PFS) of 8 months. Median overall survival (OS) was 12.3 months, and 84% (46/55) of patients died within 3 years after their initial diagnosis.^1^

Classification of ETMR as a distinct clinical entity has been an evolving process. In 2009, Li et al. reported the amplification of C19MC in a variety of tumors including not only embryonal tumor with abundant neuropil and true rosettes but also medulloepithelioma, and ependymoblastoma, suggesting that these tumors may represent different manifestations of a single biologic entity.^4^ The 2016 WHO classification of CNS tumors unified this group of C19MC-amplified tumors under the nomenclature of ETMR.^5^ While C19MC amplification is present in approximately 90% of ETMR, a small subset exists without this distinctive molecular signature. The WHO classifies these non-C19MC–amplified tumors as ETMR NOS.

Due to the rarity of ETMR and its recent classification, no large clinical investigations have been conducted to determine optimal therapy for these tumors. Horwitz et al. published a case series describing the French experience with ETMR and provided analysis of 30 patients treated according to the primitive neuroectodermal tumors of high risk (PNET-HR) protocol.^2^ On multivariate analysis, gross-total resection (GTR), radiotherapy (RT), and high-dose chemotherapy with autologous stem cell rescue (HDCT/SCR) were associated with improved outcomes. However, even with these interventions, patients had a 1-year event-free survival (EFS) and OS of only 36% and 45%, respectively.

Data from the French series suggest that our most aggressive standard therapies for pediatric brain tumors are not sufficient for cure in the vast majority of ETMR cases. New treatment approaches harnessing genomic and preclinical data defining certain therapeutic susceptibilities are therefore urgently needed. Recently, Schmidt et al. published findings from a preclinical drug screen aimed at developing novel treatment regimens for children with ETMR.^6^ An in vitro and in vivo drug screen using patient-derived ETMR cell line BT183 and its xenograft defined susceptibility to both topotecan and doxorubicin. In xenograft mice, monotherapy with topotecan or actinomycin D led to a temporary tumor response and significantly prolonged survival. Multiagent therapy with topotecan or doxorubicin in combination with methotrexate and vincristine further increased tumor responses.

A variety of infant brain tumor regimens are commonly used in the treatment of ETMR as there is no current standard of care. These approaches include the aforementioned PNET-HR protocol, the German HIT protocols, U.S. cooperative group protocols CCG99703 and ACNS0334, as well as the Head Start protocols. While there is some variability between these individual protocols, they similarly incorporate platinum and etoposide-based induction therapy with or without cyclophosphamide, vincristine, and high-dose methotrexate. Of note, none of the above infant brain tumor protocols utilize the most active agents discovered in the German drug screen. The DFCI-IRS-III for atypical teratoid rhabdoid tumor (AT/RT), however, does incorporate the use of both doxorubicin and actinomycin D into a platinum-based induction backbone. The IRS-III regimen has been investigated for use in AT/RT in a phase II study of 20 patients which included intrathecal treatments and focal radiation for children under 3 years of age. The reported 2-year PFS and OS outcomes from this study were 53% and 70%, respectively.^7^

## Materials and Methods

In this case series, we present 5 children with ETMR who received treatment using an IRS-III-based chemotherapy backbone (Table 1). The children were treated consecutively by the authors as they presented across their 4 respective institutions over a period of 6 years. No other children with newly diagnosed ETMR were treated by the authors during this period and through data closure. Patients received individualized therapy at the discretion of their primary oncologist. Caregivers gave informed consent for clinical treatment described in the series. The retrospective chart review of these cases received institutional review board approval with a waiver of informed consent.

**Table 1.** Summary of Case Series

Case	Age	Sex	Tumor Location	M Stage	C19MC	Surgery	IRS-III Treatment	HDC + ASCT	RT	Additional Therapy	EFS	OS	Status
1	32 months	F	R Parietal	M0	Positive	GTR	51 weeks	No	Focal RT	None	7 years 6 months	7 years 6 months	Alive, NED
2	39 months	F	R Parietal	M0	Positive	GTR	19 weeks	No	Focal Proton	Omburtamab DFMO	3 years 3 months	3 years 3 months	Alive, NED
3	5 months	F	R Posterior Fossa	M0	Negative	STR, then GTR	12 weeks	Yes	None	DFMO Everolimus IT Topotecan	3 years 2 months	3 years 2 months	Alive, NED
4	22 months	M	R Parietal	M0	Positive	GTR	12 weeks	No	Focal Proton	DFMO Vorinostat Topotecan Lorlatinib Irinotecan Olaparib Veliparib RT × 2	4 months	1 years 10 months	Alive, NED
5	26 months	F	R Frontal/Parietal	M0	Positive	GTR	50 weeks	No	Focal Proton	None	1 year 7 months	1 year 7 months	Alive, NED

DFMO, difluromethylornithine; F, female; GTR, gross-total resection; HDC + ASCT, high-dose chemotherapy plus autologous stem cell rescue; M, Male; NED, no evidence of disease; RT, radiotherapy; STR, subtotal resection.

## Results

### Case 1

**Figure 1.**
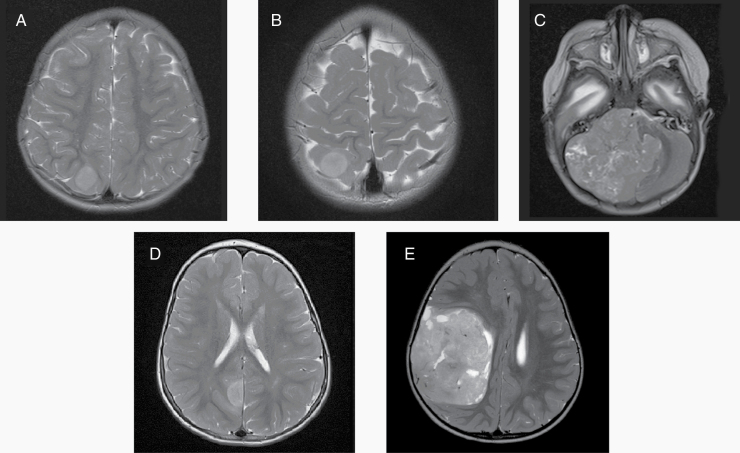
Representative MRI studies of patients. (A) A T2-weighted image of the brain from Case 1, demonstrating a hyperintense mass in the right parietal cortex. (B) A T2-weighted image of the brain from Case 2, demonstrating a lesion in the right superior parietal lobe. (C) A T2-weighted image of the brain from Case 3, demonstrating a large mass of the right posterior fossa. (D) A T2-weighted image of the brain from Case 4, demonstrating mass involving the right posterior parietal lobe. (E) A T2-weighted image of the brain from Case 5, demonstrating a large right fronto-parietal mass with midline shift.

Case 1 is a 32-month-old female who presented with a left focal seizure. An MRI of the brain revealed a 17-mm nonenhancing, T2 hyperintense mass in the right parietal cortex (Figure 1). The MRI of the spine was negative for metastatic disease. The tumor was completely resected, and pathologic examination revealed areas of closely packed embryonal cells with hyperchromatic nuclei, vesicular chromatin, and prominent nucleoli arranged around blood vessels with occasional rosettes identified. Cells were admixed with neuropil, with cortical and leptomeningeal infiltration. The tumor was negative for GFAP, LCA, CD3, and CD20. Neuropil swathes were strongly neurofilament and weakly synaptophysin positive. Ki-67 staining was 80%–90%. The tumor tissue demonstrated C19MC amplification by fluorescence in situ hybridization (FISH) (5–10 copies). CSF cytology was negative for malignant cells.

The patient began chemotherapy according to the DFCI-IRS-III chemotherapy regimen. She received 6 weeks of induction chemotherapy, followed by 6 weeks of chemoradiotherapy that included focal RT to the primary tumor bed. After finishing 6 weeks of postradiotherapy induction treatment, the patient completed an additional 26 weeks of maintenance therapy, concluding with 7 weeks of doxorubicin-containing continuation therapy.

The patient is currently 7 years and 6 months from diagnosis without evidence of tumor recurrence. She is healthy with normal vision, hearing, renal, and cardiac function. Neuropsychological evaluations reveal low average to average nonverbal measures of intellectual functioning. She is demonstrating average academic performance in school and does not require additional assistance.

### Case 2

Case 2 is a female who presented at 39 months of age with a seizure. An MRI of the brain revealed a well-circumscribed, diffusion-restricted lesion in the right superior parietal lobe (Figure 1), and the MRI of the spine was negative for metastatic disease. The patient underwent a GTR of the mass. Histological evaluation of the tumor revealed a primitive high-grade neoplasm, with LIN28A overexpression in the majority of cells by immunohistochemistry, consistent with the diagnosis of ETMR. FISH analysis confirmed amplification of the C19M/MIR-371–373 region. CSF cytology was negative for malignant cells.

The patient received 19 weeks of chemotherapy, including focal irradiation as per the DFCI-IRS-III chemotherapy regimen. Following completion of up-front chemotherapy, the patient received experimental treatment with omburtamab (8H9 monoclonal antibody linked to I131).^8^ Additional maintenance therapy with difluoromethylornithine (DFMO) was initiated and continued for 2 years.

The patient is currently 39 months from diagnosis without evidence of tumor recurrence. She is clinically well with normal vision, hearing, renal, and cardiac function. She is performing well academically in the first grade.

### Case 3

Case 3 is a female who presented at 5 months of age with vomiting and somnolence. An MRI of the brain revealed a large calcified and heterogeneously enhancing mass of the right posterior fossa measuring 6.5 × 6.5 × 4.4 cm (Figure 1). The MRI of the spine did not reveal any metastatic disease. The patient underwent a subtotal resection, with residual tumor measuring 4.9 × 4.2 × 4.3 cm, causing mass effect upon the right posterior thalamus and midbrain. Histopathology was consistent with ETMR and was confirmed by a second opinion. The tumor tissue tested positive for LIN28A by immunohistochemistry, and the 19q13.42 locus was negative for amplification by FISH. The tumor did, however, cluster with other ETMR on methylation profiling, confirming the diagnosis. Genomic sequencing of the tumor was negative for a DICER1 mutation. CSF cytology was negative for malignant cells.

The patient received 4 cycles of induction chemotherapy as per the DFCI-IRS-III regimen. The second cycle of chemotherapy was complicated by the development of sinusoidal obstructive syndrome, which responded quickly to defibrotide therapy. An MRI following the fourth chemotherapy cycle showed a 72% decrease in the size of the tumor to 4.5 × 2.4 × 2.3 cm. The patient underwent a GTR of her remaining tumor. Pathology demonstrated a significant change from the initial specimen with areas of extensive maturation and advanced neuronal/ganglionic differentiation.

The patient then received HDCT/SCR with a carboplatin and thiotepa conditioning regimen. Due to the patient’s young age, radiotherapy was deferred. She then received maintenance chemotherapy for 18 months with DFMO, everolimus, and intrathecal topotecan.

The patient is currently 38 months from diagnosis and 12 months from completion of therapy without evidence of tumor recurrence. The child is healthy with normal vision, renal, and cardiac function. She has bilateral hearing loss requiring hearing aids. She is receiving early intervention for motor and speech delays and is attending preschool.

### Case 4

Case 4 is a male who presented at 2 years of age with a left-sided seizure and hemiparesis. Brain MRI revealed a 2-cm T2 FLAIR hyperintense focus with decreased diffusivity in the right posterior parietal lobe. The MRI of the spine was negative for metastatic disease (Figure 1). The patient underwent a GTR of the tumor. Pathology revealed an embryonal tumor with neurocytic differentiation. Microarray analysis showed C19MC alteration, consistent with ETMR. CSF cytology was negative for malignant cells.

The patient began therapy according to the DFCI-IRSIII regimen, with substitution of intrathecal topotecan in place of methotrexate, hydrocortisone, and cytarabine. After 4 cycles of induction chemotherapy, disease recurrence was noted in the primary surgical cavity on surveillance imaging. He underwent a second complete resection followed by focal proton beam RT and experimental therapy with DFMO and vorinostat. After 2 months, the patient experienced a second local recurrence after which he underwent repeat GTR and was treated with focal re-irradiation and molecularly guided chemotherapy with lorlatinib and topotecan. Six months later, a third recurrence with a metastatic frontal lobe lesion was discovered and was treated with additional radiation followed by targeted therapy. He is now 22 months from diagnosis and 4 months from his last radiotherapy. He is receiving therapy with irinotecan and a PARP inhibitor and has no evidence of disease.

### Case 5

Case 5 is a female who presented at 26 months of age with progressive lethargy and vomiting. An MRI of the brain revealed a nonenhancing 8 × 6 cm right fronto-parietal mass with midline shift (Figure 1). The MRI of the spine and CSF cytology showed no evidence of metastatic spread. The patient underwent GTR of the tumor. Pathology was consistent with ETMR, including LIN28 overexpression by immunohistochemistry and C19MC (19q13.42) amplification by FISH.

The patient received therapy as per the DFCI-IRS-III chemotherapy regimen, including 6 weeks of induction chemotherapy. The patient received 6 weeks of chemoradiotherapy including focal proton beam RT to the tumor bed (54 Gy). Due to concern for toxicity, intrathecal chemotherapy was not given on Day 1 of the chemoradiotherapy cycle, as prescribed. Additionally, vincristine was held after Week 13 of therapy due to Grade 3 peripheral sensory and motor neuropathy that did not improve with 50% dose reduction in Week 10.

The patient completed an additional 26 weeks of maintenance therapy followed by 6 weeks of doxorubicin-containing continuation therapy. The final cycle of doxorubicin continuation was held due to delayed count recovery and parental preference. She received initial intrathecal treatment with methotrexate, hydrocortisone, and cytarabine but intrathecal therapy was changed to topotecan after cycle 1. The patient is currently disease-free 19 months from diagnosis and 8 months from completion of therapy.

## Discussion

ETMR is a highly aggressive, poor-prognosis pediatric CNS tumor for which there is no current standard therapy. To date, medical literature pertaining to ETMR is comprised entirely of retrospective data from patients who have received a heterogeneous group of therapies. These series suggest that GTR, RT, and HDCT/SCR may improve outcomes.^1,2,9^ However, survival for patients remains very poor even with these interventions. New biologically based approaches to the treatment of ETMR must be explored to change the trajectory of this deadly tumor.

The presented case series details the treatment of 5 children with ETMR treated according to the DFCI-IRS-III-based approach (Table 2). Four patients (80%) have PFS of more than 18 months, more than double the 8-month median survival described in the Korshunov series.^1^ These results also appear promising when compared with the French series, which described a 1-year EFS of 36%.^2^ While no definitive conclusions regarding the efficacy of the IRS-III-based chemotherapy backbone can be drawn from this small cohort, the outcomes of these patients are far better than would be expected based on historical outcomes for this tumor, and further investigation of this approach is warranted.

**Table 2.** Schematic of IRS-III Induction Chemotherapy

Week 1	2	3	4	5	6	7	8	9	10	11	12	13	14	15	16	17	18
V	V	V	V	V	V	V	V	V	V	V	V	V			V		
P			P			P			P								
D			D									D					
C						C*			C*			C			C		
			E			E			E								
															A		
I	I		I			I						I					

V = Vincristine 2 mg/m^2^ i.v. on Day 1; P = Cisplatin 90 mg/m^2^ i.v. on Day 1; D = Doxorubicin 30 mg/m^2^/day infused continuously over 48 h on Days 2 and 3; C = Cylophosphamide 300 mg/m^2^/day infused continuously over 72 h on Days 2, 3, and 4; C* = Cylophosphamide 600 mg/m^2^ i.v. on Day 2; E = Etoposide 100 mg/m^2^ i.v. on Days 1, 2, and 3; A = Actinomycin D 1.2 mg/m^2^ i.v. on Days 1, 2, 3, 4, and 5; I = Methotrexate 15 mg/m^2^, Hydrocortisone 30 mg/m^2^, Cytarabine 60 mg/m^2^ intrathecally on Day 1.

The age at presentation for the children in this series ranges from 5 to 39 months, consistent with the published median age at presentation of 2 years.^2,6^ Four patients were female, also consistent with the 2:1 female predominance noted for the tumor.^2,6,10^ Four patients’ tumors were positive for C19MC amplification, while Case 3 was negative by FISH but confirmed as ETMR by methylation profiling. Approximately 10% of ETMR are C19MC nonamplified.^11,12^ These tumors have a predisposition for the posterior fossa, consistent with the tumor location of Case 3 (Figure 1). Molecular analysis has shown that ETMRs without C19MC amplification display a similar miRNA pattern to those with C19MC amplification, including expression of C19MC miRNA, and there is no evidence to suggest that these tumors exhibit a different clinical behavior.^12^

GTR was achieved for all patients, including GTR for 4 at diagnosis and GTR for 1 after second-look surgery. None of the patients had metastatic disease at presentation. Four patients received focal RT early in the course of their treatment, with the youngest patient instead receiving consolidation with HDCT/SCR to avoid the severe cognitive effects of RT in infants. Chemotherapy was reasonably tolerated by all 5 patients with expected side effects of cytopenias and mucositis noted in all patients, some requiring transient dose reduction or chemotherapy delay. Notable adverse events included the development of mild sinusoidal obstructive syndrome in Case 3, which reversed quickly with defibrotide intervention, and the development of Grade 3 peripheral sensory and motor neuropathy in Case 5, which lead to eventual discontinuation of vincristine. The patients are exhibiting good functional status and quality of life following completion of their therapy. They are attending school and receiving supportive services as needed.

Patients in the series received interventions other than the modified IRS-III backbone, and thus their positive outcomes cannot be solely attributed to the chemotherapy agents that were administered. Assessing the specific role of preclinically active chemotherapy in these patients is somewhat challenging. Doxorubicin and actinomycin D are very large molecules with limited blood–brain barrier penetration. We hypothesize that abnormal vasculature in these high-grade tumors and disruption of the blood–brain barrier from neurosurgical resection may allow these agents to penetrate tumor tissue.^13^ This concept is supported by the successful use of these agents for the treatment of AT/RT, another aggressive infant brain tumor.^7^ Cases 1, 2, and 5 all underwent an initial GTR with no postoperative evidence of disease, preventing an assessment of objective response to treatment. Case 3, however, had a relatively large residual mass that responded to 4 cycles of DFCI-IRS-III chemotherapy with a 72% tumor reduction, enabling an eventual complete resection.

Limitations of this case series include small patient numbers as well as heterogeneity in the overall treatments administered. Cases 2 and 3 received maintenance therapy following IRS-III chemotherapy, and it is unclear how these additional agents may have contributed to these patients’ positive outcomes. Both received 18 months of treatment with DFMO, an enzyme-activated inhibitor of ornithine decarboxylase (ODC), which is the rate-limiting enzyme for polyamine biosynthesis. Polyamines, regulated by ODC, modulate eIF-5A, which is a direct regulator of the LIN28/let-7 axis.^14^ Case 2 received omburtamab, a murine IgG1 monoclonal antibody, in an attempt to deliver radioimmunotherapy through the CSF and avoid craniospinal irradiation, and Case 3 received intrathecal topotecan for CSF prophylaxis and everolimus as a targeted approach to downregulate mTOR signaling.^8,15^

To date, no prospective clinical trials specific to ETMR have been performed. Rarity of this tumor presents a significant challenge for patient accrual, and the paucity of clinical data limits the institution of a best practice. In 2019, an international panel of pediatric neuro-oncologists, neurosurgeons, and researchers met with the goal of advancing therapy for ETMR. Based on the review of retrospective series, the panel deemed that the following approaches to therapy should be adopted to improve outcomes: aggressive surgical resection with the goal of achieving a GTR during either initial or second-look surgery, judicious use of RT early in treatment for age-appropriate patients with no evidence of disease, and the use of HDCT/SCR for consolidation treatment. In light of the poor outcomes with conventional infant brain tumor regimens, the antitumor activity of doxorubicin, topotecan, and actinomycin D in preclinical models, and the positive outcomes of the children in this case series, the consensus was that a chemotherapy backbone containing preclinically active agents should be incorporated into the overall strategy for a prospective trial in ETMR.

An international consensus protocol has been developed incorporating maximal safe surgical resection, induction chemotherapy with active preclinical agents, intrathecal chemotherapy, RT, and HDCT/SCR. This protocol represents the first prospective clinical investigation specific to ETMR. In addition to the primary trial centers, the consensus protocol will be made available through a global treatment registry, providing access to the regimen for all patients. This study aims to improve survival by providing aggressive, optimized therapy for ETMR and will serve as a platform to procure tumor specimens for further preclinical endeavors and to explore new biologically promising agents. The investigation will also provide valuable prospective outcome data and correlative biological studies to serve as baseline comparators for future clinical trials.
